# Construction of porous CuCo_2_S_4_ nanorod arrays via anion exchange for high-performance asymmetric supercapacitor

**DOI:** 10.1038/s41598-017-07102-1

**Published:** 2017-07-27

**Authors:** Siyi Cheng, Tielin Shi, Chen Chen, Yan Zhong, Yuanyuan Huang, Xiangxu Tao, Junjie Li, Guanglan Liao, Zirong Tang

**Affiliations:** State Key Laboratory of Digital Manufacturing Equipment and Technology, Huazhong University of Science and Technology, 1037 Luoyu Road, Wuhan, 430074 China

## Abstract

To push the energy density limit of supercapacitors, proper pseudocapacitive materials with favorable nanostructures are urgently pursued. Ternary transition metal sulfides are promising electrode materials due to the better conductivity and higher electrochemical activity in comparison to the single element sulfides and transition metal oxides. In this work, we have successfully synthesized porous CuCo_2_S_4_ nanorod array (NRAs) on carbon textile through a stepwise hydrothermal method, including the growth of the Cu-Co precursor nanowire arrays and subsequent conversion into CuCo_2_S_4_ NRAs via anion exchange reaction. The CuCo_2_S_4_ NRAs electrode exhibits a greatly enhanced specific capacitance and an outstanding cycling stability. Moreover, an asymmetric supercapacitor using the CuCo_2_S_4_ NRAs as positive electrode and activated carbon as negative electrode delivers a high energy density of 56.96 W h kg^−1^. Such superior performance demonstrate that the CuCo_2_S_4_ NRAs are promising materials for future energy storage applications.

## Introduction

Supercapacitors (SCs) have drawn great attention in the past decades due to their long life span, high power density, fast charge and discharge rate and environmental friendliness. However they still suffer from lower energy density than lithium batteries^1, 2^. Developing rational electrode nanostructures with proper materials, which allowing good conductivity, high porosity and good mechanical strength, could be a feasible way to improve the energy density of supercapacitors without sacrificing the power density and cycling stability^3, 4^. In general, supercapacitors can be classified into two major types, electrical double-layer capacitors (EDLCs) and pseudocapacitors. Compare to EDLCs, which store capacitance by the accumulation of charges on the surface of materials, pseudocapacitors could offer much higher specific capacitance due to the fast interfacial faradaic redox reactions^5^. Moreover, when integrating pseudocapacitive and EDLC electrodes into an asymmetric supercapacitor, the energy density, power density and operation voltage window can be further enhanced^6, 7^.

Transition metal oxides, hydroxides and their compounds have been widely studied for high-performance supercapacitor applications because of their high specific capacitance, low cost and great structure flexibility^8–12^. However, these materials still suffer from low electron conductivity, which could restrict their further application in practical energy storage device^13, 14^. Recently, ternary transition metal sulfides have been widely studied as promising pseudocapacitive materials due to their enhanced electrochemical performance. Ternary transition metal sulfides with enhanced electric conductivity in comparison to the single element sulfides can be ascribed to the lower band-gap energy^15^. Besides, ternary transition metal sulfides can provide richer redox reaction sites coming from the multiple counterpart elements, leading to better electrochemical activity and higher specific capacitance^16^. Moreover, the anion exchange (from oxygen to sulfur) could make the structure more flexible because of the lower electronegativity of sulfur^17^. For these reasons, extensive research efforts have been devoted to develop ternary transition metal sulfides, especially NiCo_2_S_4_, which exhibits remarkable electrochemical performance^15, 18, 19^. Nevertheless, new kinds of ternary transition metal sulfides with superior conductivity and high energy density are still pursued for further improvement of supercapacitor performance. According to previous report, CuCo_2_S_4_ is expected to be a promising electrode material candidate for supercapacitor applications^20, 21^. Designing porous electrode micro/nanostructures with large surface area are also important for efficient electrolyte ion diffusion, which could provide more electroactive sites for faradaic redox reactions. Among different electrode structures, one dimensional porous nanostructures with well-defined interior voids, such as nanowires, nanotubes and nanorods, have attracted enormous research interests in supercapacitor applications^13, 20, 22, 23^.

As for the growth of porous ternary transition metal sulfides, most of the reported synthetic methods are based on a two-step process, including the synthesis of transition metal precursors and a subsequent anion exchange reaction^13, 16, 20^. The anion exchange process is a low-cost and effective method to transform solid precursors into porous nanostructures. Various porous transition metal sulfides nanostructures, such as NiCo_2_S_4_ nanotubes^16^, NiCo_2_S_4_ nanosheets^19^, CuCo_2_S_4_ nanoneedles^20^ and FeCo_2_S_4_ nanotubes^2^, have been fabricated by this method and show enhanced electrochemical performance. In general, the diffusion depth of the electrolyte ion into the electrode structure is about 20 nm, which means that the pseudocapacitive materials under 20 nm depth could not be utilized sufficiently^24, 25^. Porous nanorod arrays formed by interconnected nanoparticles possess various advantages, such as short diffusion path, abundant electroactive sites and enlarged contact area between the active materials and the electrolytes. However, it is still challenging to fabricate such nanostructures for high-performance supercapacitor applications.

Herein, we present a facile two-step hydrothermal method for synthesizing porous CuCo_2_S_4_ nanorod arrays (NRAs) supported on 3D flexible carbon textile for high-performance supercapacitors. The as-obtained CuCo_2_S_4_ NRAs electrodes exhibits a significantly enhanced specific capacitance and cycling stability. More importantly, we have successfully assembled the porous CuCo_2_S_4_ NRAs with activated carbon as a high-performance solid-state asymmetric supercapacitor. The as-fabricated CuCo_2_S_4_ NRAs//AC asymmetric supercapacitor delivers a high energy density of 56.96 W h kg^−1^ at the power density of 320 W kg^−1^ and an outstanding cycling performance by retaining 88% of initial capacitance after 5000 cycles.

## Results and Discussion

**Figure 1:**
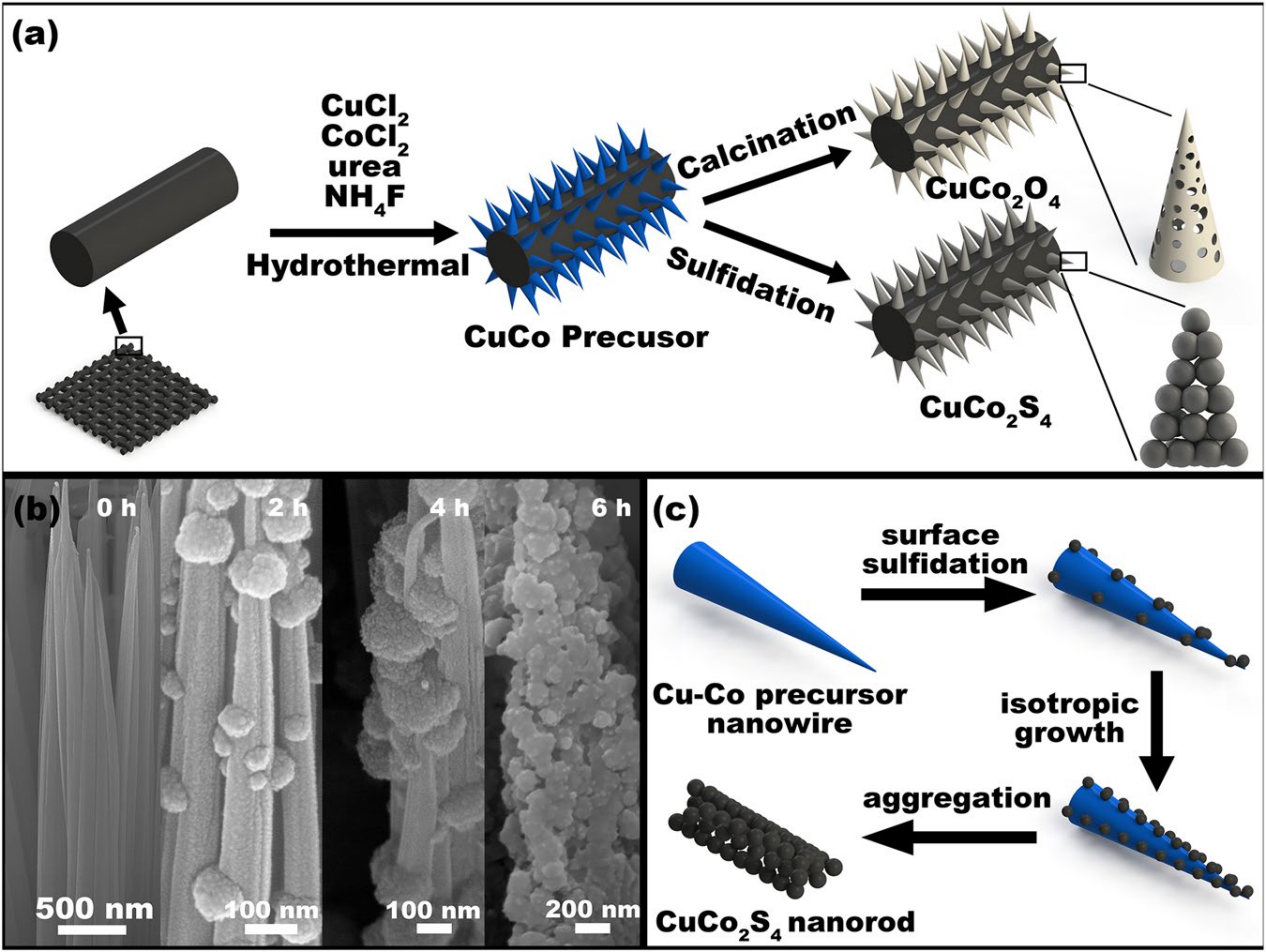
(**a**) Schematic illustration of the synthetic process of the CuCo_2_O_4_ NWAs and the CuCo_2_S_4_ NRAs on the carbon textile. (**b**) SEM images of the obtained samples over different sulfidation time. (**c**) Schematic illustration of the formation mechanism of the CuCo_2_S_4_ NRAs.

Figure 1a illustrates the formation of porous CuCo_2_S_4_ nanorod arrays (NRAs) on 3D carbon textile through a two-step approach. Firstly, Cu-Co precursor nanowire arrays directly grew on carbon microfibers under hydrothermal condition. Then, the precursor nanowire arrays transformed into the CuCo_2_S_4_ NRAs on the basis of an anion exchange reaction (O^2−^ to S^2−^). The formation of CuCo_2_S_4_ NRAs from Cu-Co precursors could be demonstrated by the dissolution and nucleation process. During this process, divalent sulfur ions (S^2−^) ions released from the decomposition of thioacetamide (TAA) initially reacted with Cu-Co precursors on the surface to form CuCo_2_S_4_ nanoparticles, then these particles aggregated along the nanowire direction. As the reaction went on, the amount of aggregated CuCo_2_S_4_ nanoparticles increased while the amount of precursor nanowires decreased and finally disappeared. As a result, the solid precursor nanowire arrays turned into porous CuCo_2_S_4_ NRAs. This transformation process is confirmed by the SEM images of the obtained samples over different sulfidation time under the hydrothermal environment (Fig. 1b). The anion exchange is an isotropic process, so the overall morphologies of the resultant CuCo_2_S_4_ NRAs is analogous to the precursor nanowire arrays. The anion exchange process is briefly illustrated in Fig. 1c. Besides, the CuCo_2_O_4_ nanowire arrays (NWAs) were transformed from the Cu-Co precursor nanowire arrays through a direct calcination process, which is also demonstrated in Fig. 1a.

**Figure 2:**
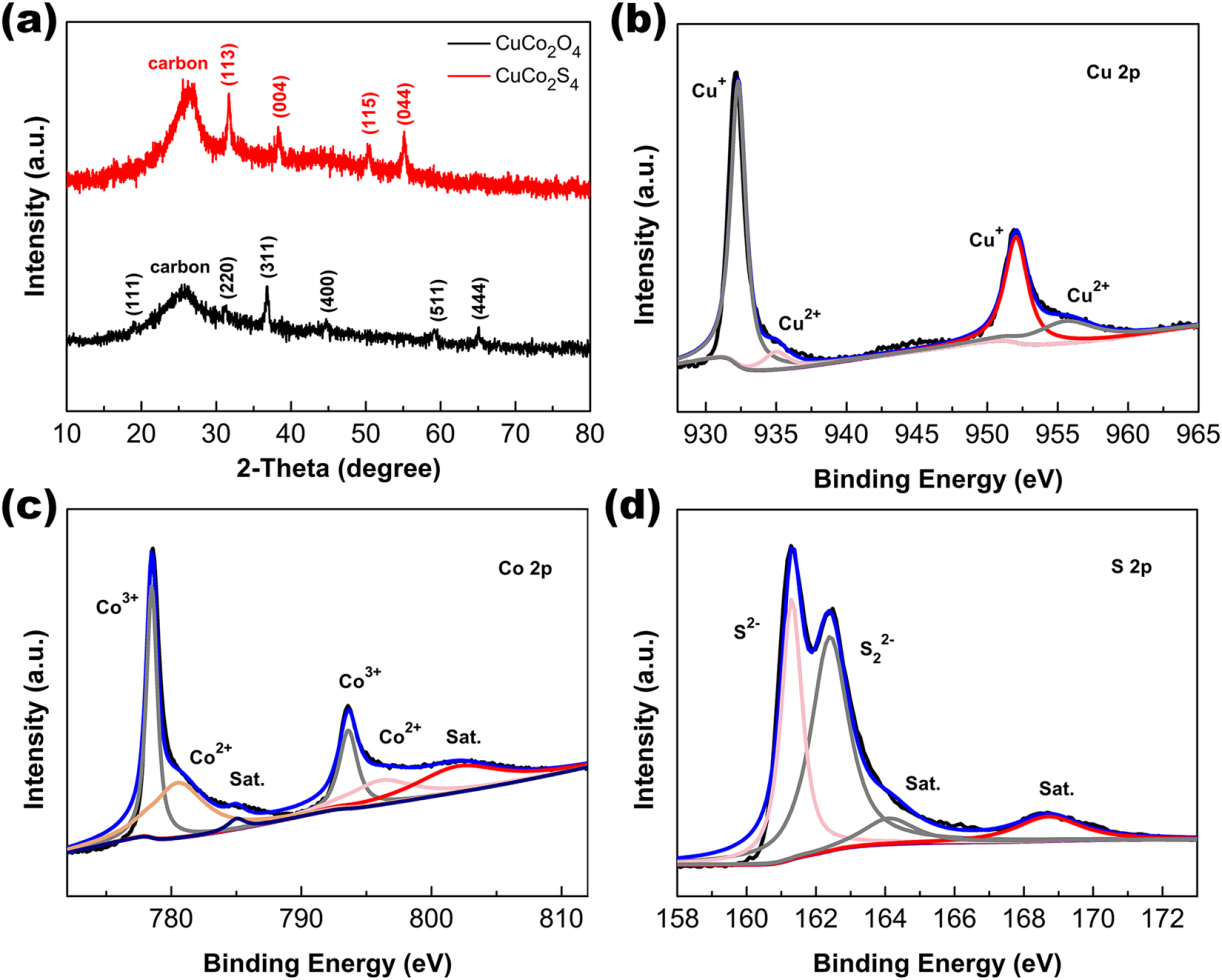
(**a**) XRD patterns of the CuCo_2_O_4_ NWAs and CuCo_2_S_4_ NRAs on carbon textiles. Typical element XPS spectra of the resultant CuCo_2_S_4_ NRAs. (**b**) Cu 2p, (**c**) Co 2p, (d) S 2p.

The XRD patterns of the CuCo_2_O_4_ NWAs and CuCo_2_S_4_ NRAs on carbon textiles are shown in Fig. 2a. The broad peaks at 26.3° in the patterns arise from the carbon textile substrates. The diffraction peaks at 19.1°, 31.4°, 36.9°, 45.1°, 59.6° and 65.7° collected from the CuCo_2_O_4_ NWAs can be well indexed with spinel phase of CuCo_2_O_4_ (JCPDS Card No. 1–1155)^26, 27^. Then, porous CuCo_2_S_4_ NRAs were chemically transformed from the Cu-Co precursor nanowire arrays via an anion exchange reaction. As can be seen in Fig. 2a, the CuCo_2_S_4_ NRAs reveal the diffraction peaks at 2-theta values of 31.3°, 37.9°, 50.0° and 54.8°, which can be well identified as (113), (004), (115) and (044) planes (JCPDS Card No. 42–1450), respectively^17, 28^. The broadened diffraction peaks with low intensity indicate the poor crystallization of the CuCo_2_S_4_ NRAs. X-ray photoelectron spectroscopy (XPS) test was carried out to further investigate the chemical bonding state of the CuCo_2_S_4_ NRAs after the anion exchange reaction. The survey spectra of the CuCo_2_S_4_ NRAs shown in Fig. S1 indicates the presence of Cu, Co, and S elements. Figure 2b–d show the high-resolution Cu 2p, Co 2p and S 2p XPS spectra and the corresponding fits by Gaussian method. The Cu spectrum (Fig. 2b) is well fitted with two spin-orbit doublets, which are characteristics of Cu^+^ and Cu^2+^, respectively^29^. The binding energies at around 932.3 and 952.1 eV of the Cu 2p spectra can be assigned to Cu^+^ while the binding energies at 935 and 955.6 eV to Cu^2+^. Two kinds of cobalt oxidation states can be detected in the Co 2p spectrum (Fig. 2c), where one doublet corresponding to Co^2+^ located at 778.5 and 793.6 eV with a splitting value of 15.1 eV, another corresponding to Co^3+^ at about 780.5 and 796.1 eV with a splitting value of 15.6 eV^30^. In the S 2p spectrum (Fig. 2d), the binding energy centered at 161.3 eV and 162.4 eV can be assigned to S^2−^ and S_2_^2−^, respectively. Besides, two broad peaks detected at 164.1 and 168.7 eV reveal the existence of sulphate^29, 31, 32^. In general, the chemical composition of the CuCo_2_S_4_ NRAs is confirmed by the XRD and XPS results. The EDS spectrum shown in Fig. S2 demonstrates the existences of Cu, Co and S elements in the CuCo_2_S_4_ NRAs. The element molar ratio of Cu, Co and S is 1:2.18:3.69, further confirming the formation of pure CuCo_2_S_4_. Figure S3 shows the N2 adsorption-desorption isotherm and pore size distribution curve of the as-synthesized porous CuCo_2_S_4_ NRAs. The typical type IV isotherm with the H3 hysteresis loop (Fig. S3a) reveal the mesoporous nature of the CuCo_2_S_4_ NRAs. According to the multi-point BET equation, specific surface area of the CuCo_2_S_4_ NRAs is 64.117 m^2^ g^−1^. The pore size distribution of the CuCo_2_S_4_ (Fig. S3b) calculated from adsorption data using BJH model shows a two peaks centered at 2.0 and 4.9 nm, respectively. These results demonstrate that the CuCo_2_S_4_ NRAs have large surface area and high porosity, which could enrich the electroactive sites and facilitate the diffusion of electrolyte ions.

**Figure 3:**
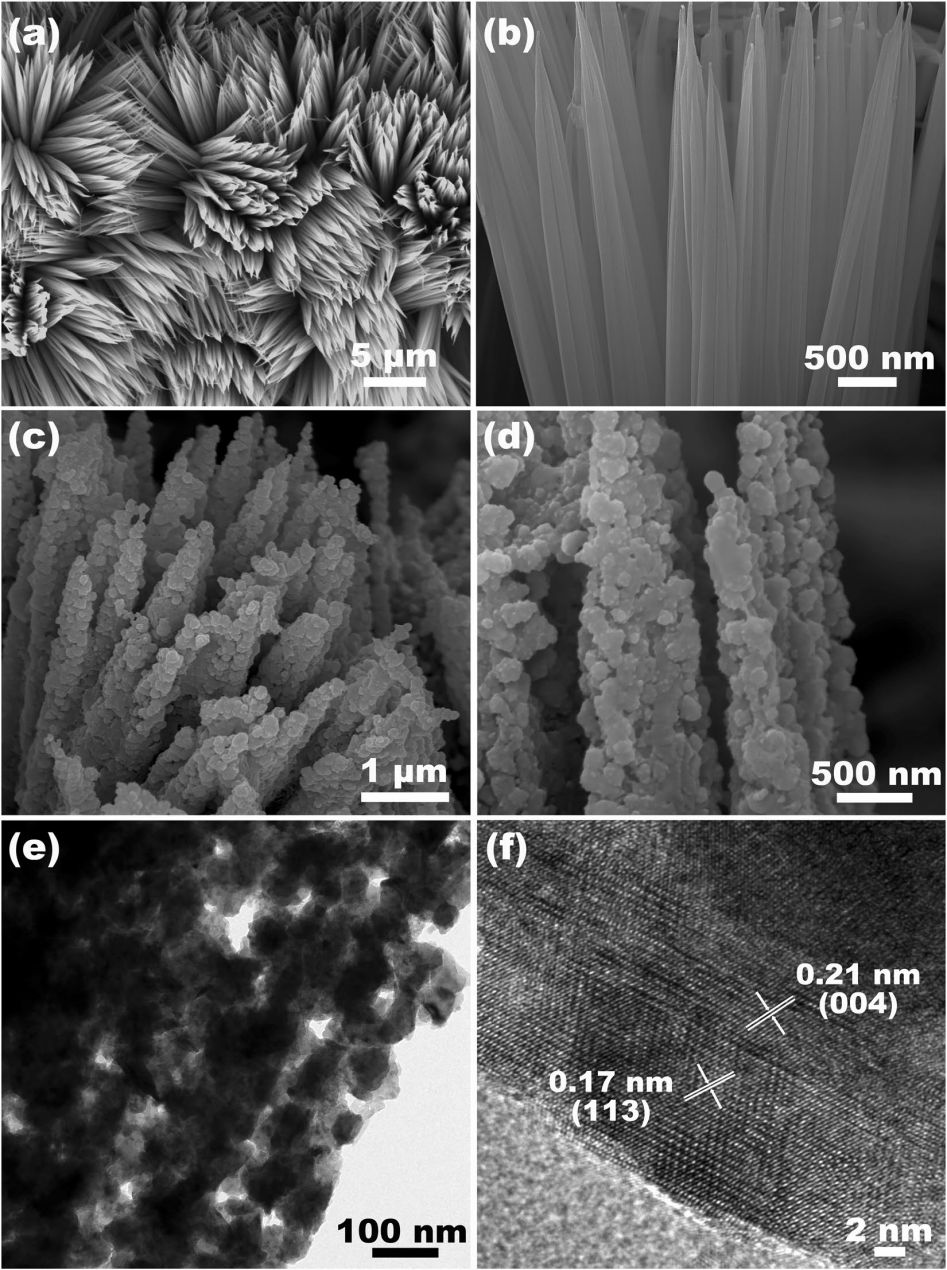
FESEM images of (**a**,**b**) the Cu-Co precursor nanowire arrays and (**c**,**d**) the CuCo_2_S_4_ NRAs on carbon textiles at different magnifications, (**e**) TEM and (**f**) HRTEM image of the CuCo_2_S_4_ NRAs scratched from carbon textile.

Figure 3a–d presents the morphology of the as-obtained Cu-Co precursor NWAs and CuCo_2_S_4_ NRAs. As can be seen from the SEM images in Fig. 3a, the needle-like Cu-Co precursor NWAs are uniformly grown on carbon microfiber. From the enlarged view (Fig. 3b), the bunched structure of the Cu-Co precursor NWAs can be obviously observed, which could provide stable backbone for the formation of the CuCo_2_S_4_ NRAs. The abundant void space between the nanowires could also facilitate the accessible of sulfur ions during the anion exchange process. After the anion exchange reaction, the Cu-Co precursor NWAs were completely turned to the CuCo_2_S_4_ NRAs. Figure 3c clearly reveals the bunched structure of the CuCo_2_S_4_ NRAs. The overall morphology was maintained after the sulfidation process. As shown in Fig. 3d, the CuCo_2_S_4_ NRAs composed of nanoparticles subunits are thick and porous, which can be further confirmed by TEM image in Fig. 3e. The dark granular area corresponds to the interconnected particles formed CuCo_2_S_4_ NRAs. The abundant void space provided by the CuCo_2_S_4_ NRAs can be effective to increase the specific surface area and facilitate the penetration of electrolyte. The nanorod is polycrystalline due to the diffractions of many randomly oriented and interconnected nanoparticles, which is confirmed by the HRTEM image in Fig. 3f. The observed two sets of lattice spacings are measured to be 0.17 nm and 0.21 nm, corresponding to the (113) and (004) planes of CuCo_2_S_4_, respectively, coinciding well with the above XRD results.

**Figure 4:**
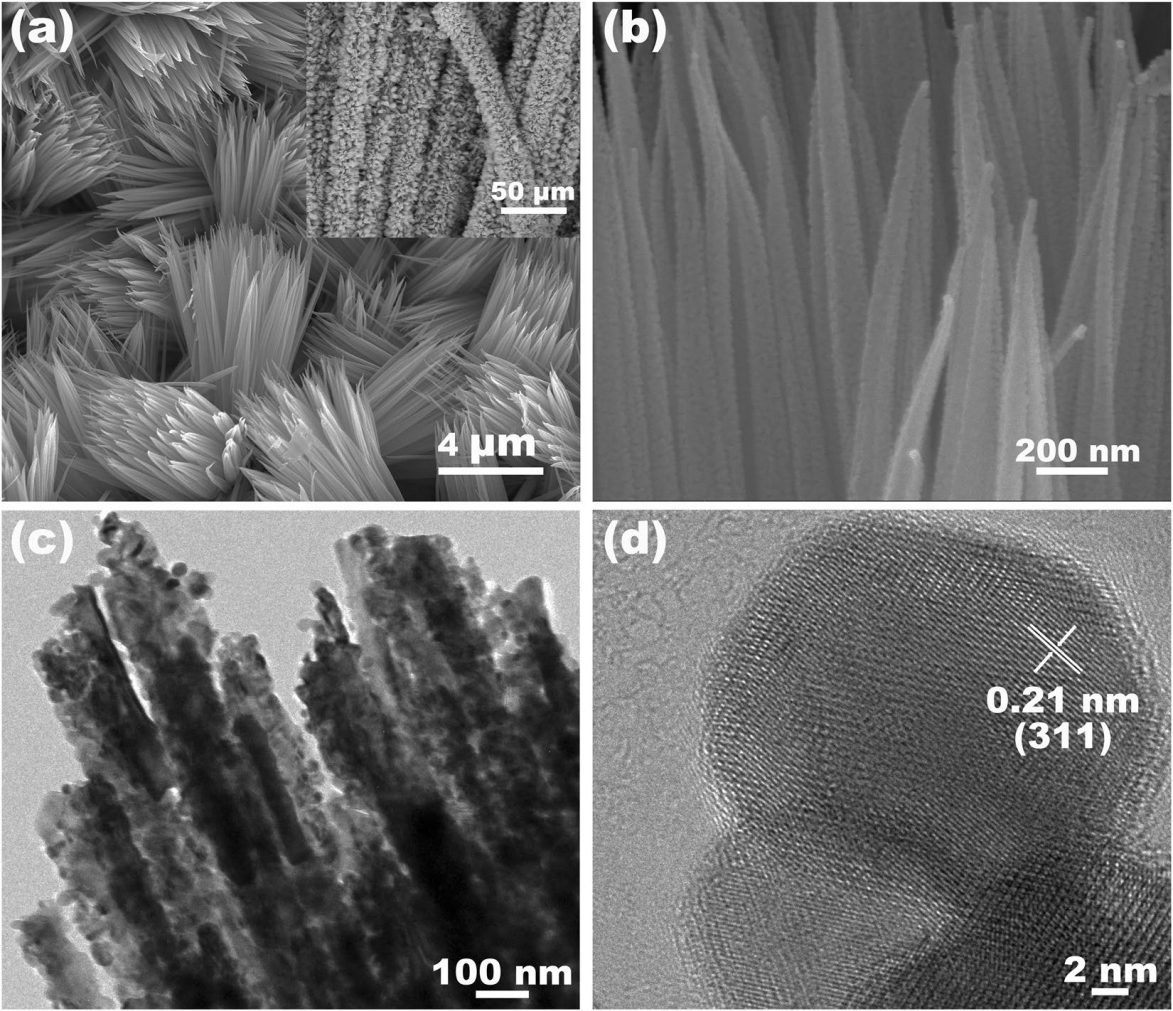
(**a**,**b**) FESEM images of the CuCo_2_O_4_ NWAs on carbon textile at different magnifications. (**c**) TEM and (**d**) HRTEM image of the CuCo_2_O_4_ NWAs.

In comparison, the mesoporous CuCo_2_O_4_ NWAs were also prepared via directly annealing the Cu-Co precursor nanowire arrays on carbon textile at 350 °C in air for 2 h. Figure 4a,b show the low and high magnification SEM images of the CuCo_2_O_4_ NWAs. The carbon fiber is uniformly covered by the CuCo_2_O_4_ NWAs on a large scale (the inset of Fig. 4a). Higher magnification SEM image in Fig. 4b shows mesoporous structure of the CuCo_2_O_4_ NWAs. The formation of these nanopores in the structure can be ascribed to gas release during the decomposition of the Cu-Co precursor nanowires^33, 34^. TEM image (Fig. 4c) further indicates the loose nanostructure consisting of conjoint nanoparticles, which is similar the CuCo_2_S_4_ NRAs. The HRTEM image (Fig. 4d) exhibits a lattice spacing of 0.21 nm, corresponding to the (311) plane of CuCo_2_O_4_.

**Figure 5:**
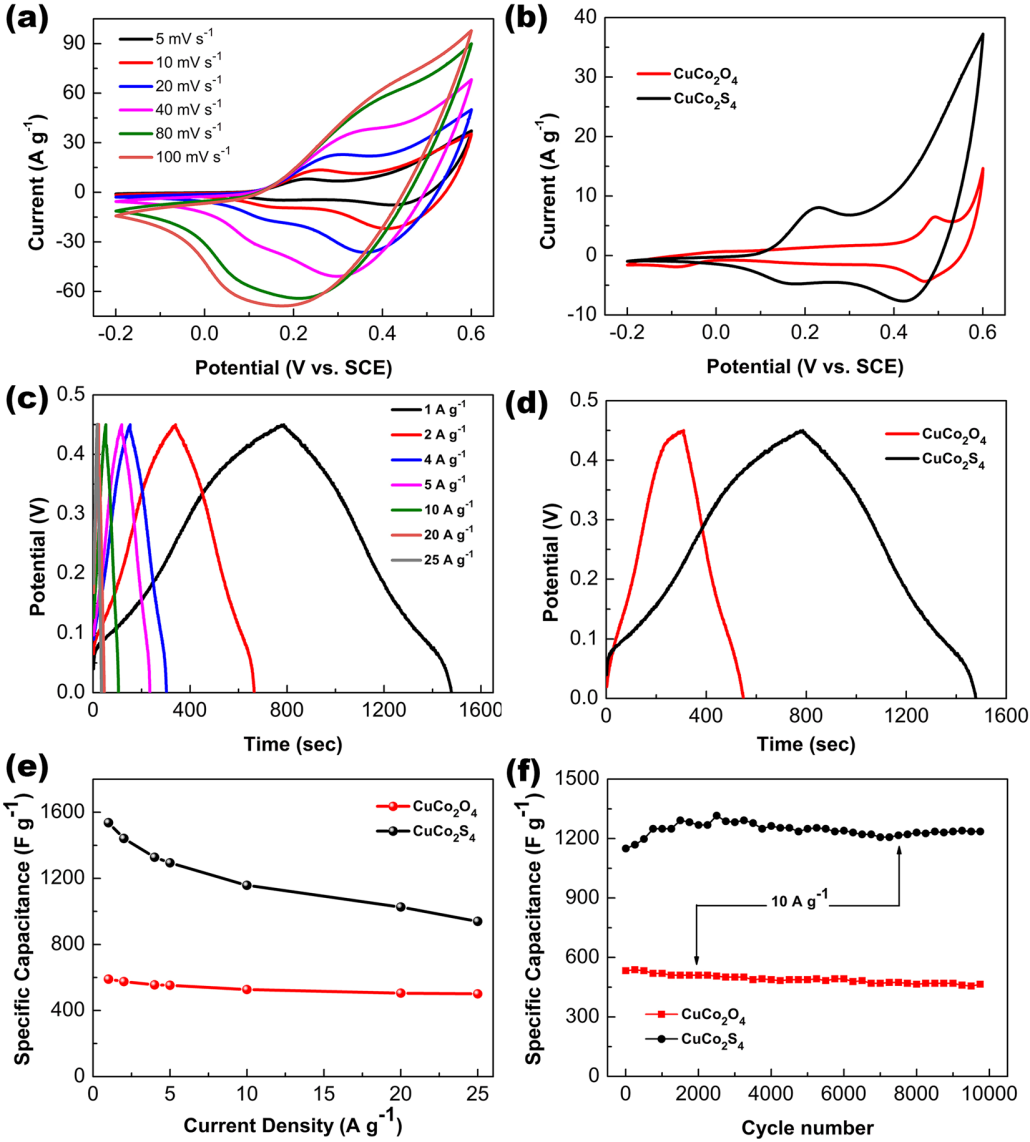
(**a**) CV and (**c**) galvanostatic charge-discharge curves of the CuCo_2_S_4_ NRAs electrode at various scan rate and current densities, (**b**) CV and (**d**) galvanostatic charge-discharge curves of the CuCo_2_O_4_ NWAs and the CuCo_2_S_4_ NRAs electrodes recorded at a scan rate of 5 mV s^−1^ and at a current density of 1 A g^−1^, (**e**) specific capacitances as a function of current densities for the CuCo_2_O_4_ NWAs and the CuCo_2_S_4_ NRAs electrodes, (**f**) cycling performance of the CuCo_2_O_4_ NWAs and the CuCo_2_S_4_ NRAs electrodes at 10 A g^−1^ for 10000 cycles.

In order to investigate the electrochemical properties of the CuCo_2_S_4_ NRAs and the CuCo_2_O_4_ NWAs electrodes, three-electrode measurements were carried out by standard cyclic voltammetry (CV) and galvanostatic charge-discharge (GCD) tests in 3 M aqueous KOH electrolyte. Figure 5a exhibits the typical CV curves of the CuCo_2_S_4_ NRAs electrode obtained at various scan rates over a potential range from −0.2 to 0.6 V. The CV curves show two pairs of clear redox peaks, especially at low scan rates, indicating the faradaic redox reactions of the CuCo_2_S_4_ NRAs. As the scan rate increases from 5 mV s^−1^ to 100 mV s^−1^, the current density increases accordingly, verifying that the CuCo_2_S_4_ NRAs electrode has good ion and electron conductivity even at high scan rate. Figure 5b presents the CV comparison for the CuCo_2_O_4_ NWAs and the CuCo_2_S_4_ NRAs electrodes recorded at a scan rate of 5 mV s^−1^. It is obvious that the curve integral area of the CuCo_2_S_4_ NRAs electrode is much larger than the CuCo_2_O_4_ NWAs electrode, indicating a higher capacitance of the CuCo_2_S_4_ NRAs electrode. Well-defined redox peaks observed in both CV curves can be mainly attributed to the faradaic redox reactions in KOH electrolyte, which reveal the pseudocapacitive characteristics of the CuCo_2_O_4_ NWAs and the CuCo_2_S_4_ NRAs electrodes. The shape of these curves are adjacent, which is possibly due to the similar redox reaction processes of Cu^2+^/Cu^+^ and Co^4+^/Co^3+^/Co^2+^, respectively^17, 35^. The electrochemical performance of the bare carbon textile substrate was also tested. As shown in Fig. S4, the curve integral area of the bare carbon textile is much smaller than the CuCo_2_O_4_ NWAs and the CuCo_2_S_4_ NRAs electrodes, confirming that the contribution of the substrate towards the overall capacity is negligible. Figure 5c shows the GCD curves of the CuCo_2_S_4_ NRAs electrode with a voltage window of 0–0.45 V (vs. SCE) at various current densities ranging from 1 to 25 A g^−1^. Distinct potential plateau regions observed in the GCD curves confirm the pseudocapacitive behaviour of the CuCo_2_S_4_ NRAs. The comparison of the GCD curves at 1 A g^−1^ between the CuCo_2_O_4_ NWAs and the CuCo_2_S_4_ NRAs electrodes is shown in Fig. 5d. Obviously, the discharge time of the CuCo_2_S_4_ NRAs electrode is much longer than the CuCo_2_O_4_ NWAs electrode, signifying the outstanding electrochemical performance of the as-synthesized CuCo_2_S_4_ NRAs via anion exchange reaction. Figure 5e demonstrates the specific capacitances of these two electrode as a function of the loading mass and current densities. The specific capacitances of the CuCo_2_S_4_ NRAs electrode are 1536.9, 1441.1, 1327.5, 1293.4, 1157.8, 1026.2 and 939.4 F g^−1^ at current densities of 1, 2, 4, 5, 10, 20 and 25 A g^−1^, respectively, which are much higher than the corresponding values of the CuCo_2_O_4_ NWAs electrode. In addition, the long-term cycling stability of the CuCo_2_O_4_ NWAs and CuCo_2_S_4_ NRAs electrodes were also investigated by the repeat GCD tests at a current density of 10 A g^−1^. The specific capacitance of the CuCo_2_S_4_ NRAs electrode maintained 1235.2 F g^−1^ after 10000 cycles, only a 5.1% drop from the maximum value, while the total capacitance loss of the CuCo_2_O_4_ NWAs electrode after 10000 cycles is around 12.8%. The cycling performance of the CuCo_2_S_4_ NRAs is better than the CuCo_2_O_4_ NWAs, and also comparable to many transition metal sulfide-based pseudocapacitive materials^36–39^. The overall morphology of the CuCo_2_S_4_ NRAs after 10000 charge-discharge cycles shows no obvious change (Fig. S5), further indicating the superior stability of the porous CuCo_2_S_4_ nanostructure. The comparison for the Nyquist plots of the CuCo_2_S_4_ NRAs and CuCo_2_O_4_ NWAs electrodes are shown in Fig. S6. The vertical lines in the low frequency zone represent the ideal supercapacitor behaviour, while the semi-circles in the high frequency zone relate to the charge-transfer resistance. Besides, the equivalent series resistances (Rs, the intercept of the real axis) correspond to the bulk resistance of the system. As can be seen, the CuCo_2_S_4_ shows smaller Rs than the CuCo_2_O_4_, which can be possibly attributed to the lower electronegativity of sulfur.

The enhancement for the pseudocapacitive performance of the CuCo_2_S_4_ NRAs can be attributed to the following aspects. Firstly, abundant void space between the porous nanostructures not only provide shorten distance for the diffusion of the electrolyte, but also offer a great deal of electroactive sites for the faradaic redox reactions, and hence improve the utilization of the pseudocapacitive materials. Secondly, the nanoscale and conductive CuCo_2_S_4_ NRAs directly grown on 3D carbon textile with superior mechanical strength and conductivity, which can facilitate the electron transportation. Finally, the bunched nanorods can serve as the physical buffer layer to suppress the structural pulverization, therefore enhancing cycling stability^2, 40^.

**Figure 6:**
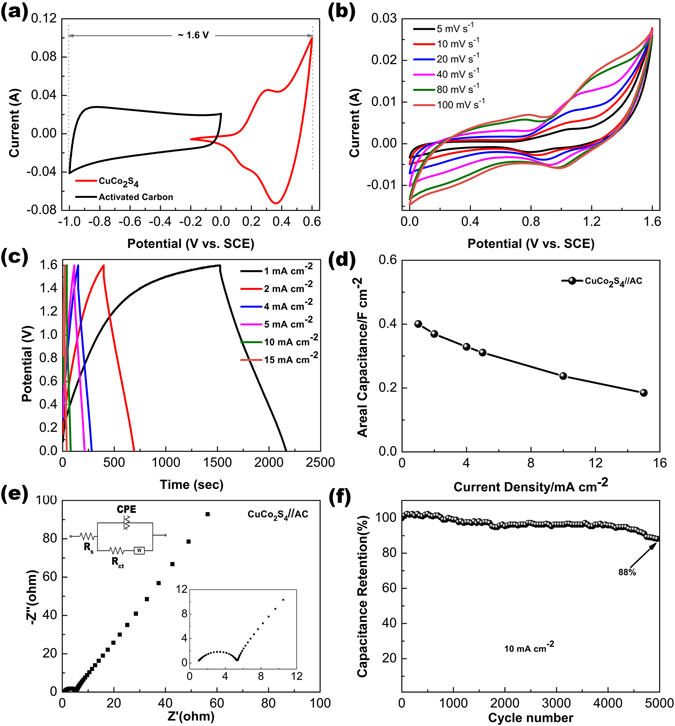
(**a**) CV curves of AC and CuCo_2_S_4_ NRAs electrodes at the scan rate of 10 mV s^−1^, (**b**) CV curves of the CuCo_2_S_4_//AC ASC at various scan rates, (**c**) GCD curves of the ASC at various current densities, (**d**) specific capacitance of the ASC as a function of current density, (**e**) Nyquist plot of the ASC recorded from 0.01 to 100 kHz at open circuit potential at open circuit potential, the insets show the equivalent circuit (top left) and enlarged Nyquist plot (bottom right) (**f**) cycling performance of the of the ASC at 10 mA cm^−2^.

To further investigate the CuCo_2_S_4_ NRAs for practical applications, a solid-state asymmetric supercapacitor (ASC) was fabricated using pseudocapacitive CuCo_2_S_4_ NRAs on carbon textile as the positive electrode, the activated carbon on carbon textile (AC) as the negative electrode and PVA/KOH as the electrolyte. The integration of the ASC device is similar to our previous work^41^. Figure 6a exhibits the CV curves of the AC and CuCo_2_S_4_ NRAs electrodes operated at voltage windows of −1.0–0 V and −0.2–0.6 V, respectively. The CV curve of the AC electrode exhibits a nearly rectangular shape without visible redox peak, demonstrating a typical EDLC behaviour. Meanwhile the CuCo_2_S_4_ NRAs electrode shows obvious redox peaks, which can be ascribed to the pseudocapacitive characteristics. Therefore, it can be concluded that such two electrodes configuration as an ASC can be operated at a voltage range of 0–1.6 V. Figure 6b shows the CV curves of the as-assembled CuCo_2_S_4_ NRAs//AC ASC at various scan rates between 0 and 1.6 V. The CV curves exhibit joint contribution of EDLC and pseudocapacitive. As the scan rate increases from 5 to 100 mV^−1^, the shapes of the CV curves remain unchanged, indicating good fast charge-discharge performance of the device. Figure 6c presents the GCD curves of the ASC device at various current densities from 1 to 15 mA cm^−2^ within the voltage window of 0–1.6 V. The areal capacitances calculated from the GCD curves are 0.40, 0.37, 0.33, 0.31, 0.24 and 0.18 F cm^−2^ corresponding to the current densities of 1, 2, 4, 5, 10 and 15 mA cm^−2^, respectively (Fig. 6d). The Nyquist plot and the corresponding equivalent circuit of the as-fabricated ASC device are exhibited in Fig. 6e. The vertical line in the low-frequency region demonstrates the Warburg resistance, which reflects the diffusion of the electrolyte ions. The suberect line in the low-frequency area indicates a pure capacitive behaviour of the ASC device. In the high-frequency region (bottom right of Fig. 6e), the low intercept at Z real axis of 1.02 Ω implies a low equivalent series resistance of the ASC device.

The repeated GCD measurement was conducted at a current density of 10 mA cm^−2^ to evaluate the durability of the as-assembled ASC device. As depicted in Fig. 6f, the capacitance increases during the initial 300 cycles, which can be ascribed to the activation process of the CuCo_2_S_4_ NRAs^42^. Then the capacitance gradually decreases due to the destruction of the electrode material after a great deal of vigorous redox reactions. Remarkably, 88% of initial capacitance of the ASC device can be maintained even after 5000 cycles.

**Figure 7:**
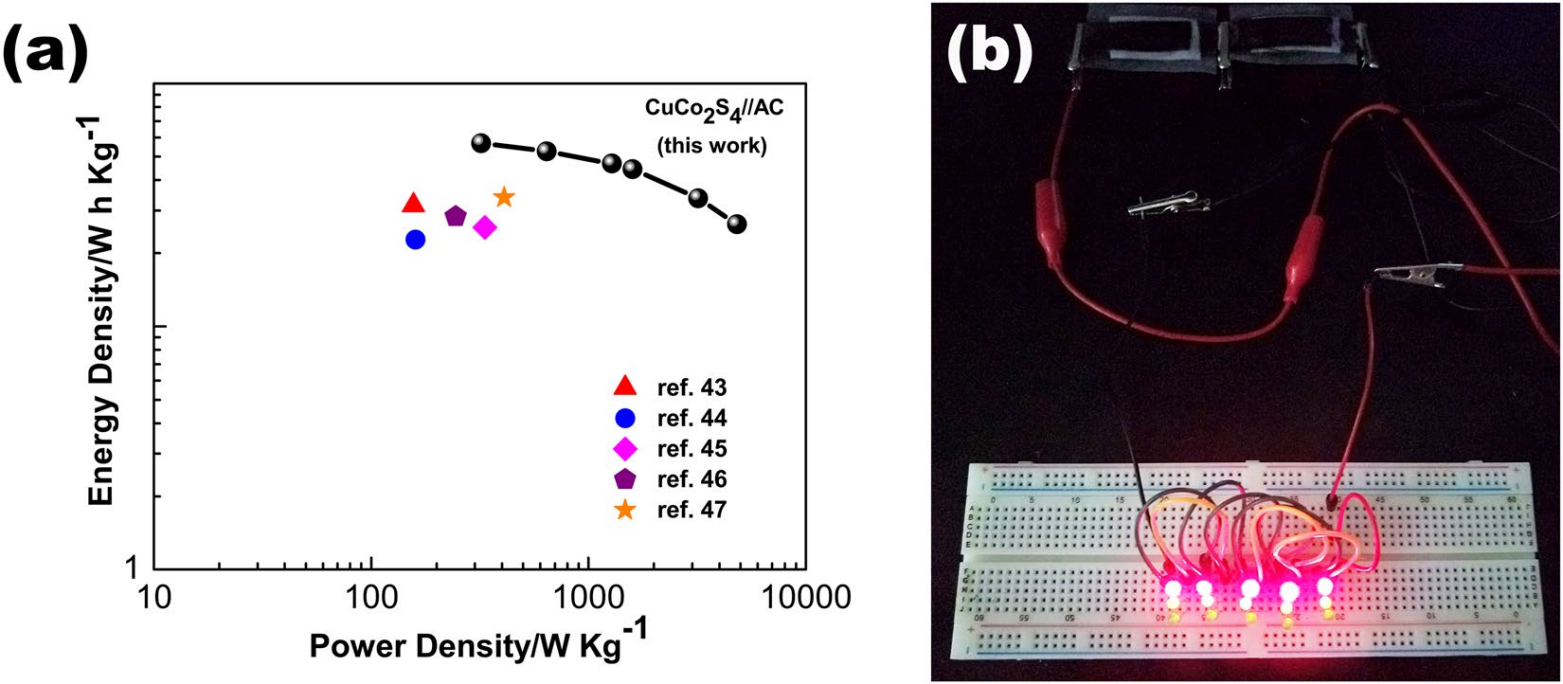
(**a**) Ragone plot of the CuCo_2_S_4_ NRAs//AC ASC, (**b**) two ASC devices connected in series can light up 15 LEDs.

It is also of great significance to investigate the energy and power densities of the ASC device for practical applications. The Ragone plots of the CuCo_2_S_4_ NRAs//AC ASC calculated from the GCD curves at different current densities. As can be seen in Fig. 7a, the ASC device achieves a maximum energy density of 56.96 W h kg^−1^ at the power density of 320 W kg^−1^. Even at a high power density of 4800 w kg^−1^, the energy density can still remain 26.29 W h kg^−1^. The energy and power densities of our CuCo_2_S_4_ NRAs//AC ASC device are also superior to those of many previous reported transition metal sulfide-based asymmetric supercapacitors, such as NiCo_2_S_4_ nanotubes//RGO ASC (31.5 W h kg^−1^ at 156.6 W kg^−1^)^43^, rod-like NiCo_2_S_4_//AC (22.8 Wh kg^−1^ at 160 W kg^−1^)^44^, mesoporous NiCo_2_S_4_ nanosheets//AC ASC (25.5 Wh kg^−1^ at 334 W kg^−1^)^45^, mesoporous NiCo_2_S_4_ nanoparticles//AC ASC (28.3 Wh kg^−1^ at 245 W kg^−1^)^46^ and CoNi_2_S_4_ nanosheet arrays//AC ASC (33.9 Wh kg^−1^ at 409 W kg^−1^)^47^. To demonstrate the practical application, 15 light-emitting diodes (LEDs) were successfully lighted up by two connected CuCo_2_S_4_ NRAs//AC ASC devices, as shown in Fig. 7b.

In summary, we have successfully synthesized porous CuCo_2_S_4_ NRAs on carbon textile via a facile two-step hydrothermal method. The mechanism of the anion exchange process is illustrated. A high specific capacitance of 1536.9 F g^−1^ at 1 A g^−1^ with an excellent cycling stability (5.1% drop from the maximum capacitance after 10000 cycles) is achieved, which can be ascribed to the porous nanostructure, rich electroactive sites and high conductivity of the bunched CuCo_2_S_4_ NRAs. The as-fabricated CuCo_2_S_4_ NRAs//AC asymmetric supercapacitor can operate at a high voltage window of 1.6 V and deliver a high energy density of 56.96 W h kg^−1^ at the power density of 320 W kg^−1^. Moreover, the ASC device shows an outstanding cycling performance by retaining 88% of initial capacitance after 5000 cycles. The practical application of our ASC device was successfully demonstrated by lighting up 15 LEDs. Such superior electrochemical performance indicate that the CuCo_2_S_4_ NRAs are promising pseudocapacitive materials for high performance supercapacitors.

## Methods

### Synthesis of CuCo_2_O_4_ NWAs and CuCo_2_S_4_ NRAs on carbon textiles

The chemicals were of analytical grade and used without further purification. Prior to synthesis, commercial carbon textiles (CeTech CO. Ltd., China; 12.5 mg cm^−2^, 0.3 mm in thickness) were cleaned by ethanol twice for 5 min in the ultrasonic cleaner. In a typical synthetic process, 0.24 g of CuCl_2_•2H_2_O, 1.03 g of CoCl_2_ • 6H_2_O, 2.16 g of urea and 0.86 g of NH_4_F were dissolved in 60 ml of DI water to form a homogeneous solution. The obtained solution was transferred into a Teflon-lined stainless steel autoclave and a piece of cleaned carbon textile was placed into the solution, then the autoclave was sealed and kept at 120 °C for 6 h. After cooled down to room temperature, the carbon textile substrate covered with the Cu-Co precursors was rinsed with DI water several times and then dried at 60 °C for 12 h.

The as-prepared Cu-Co precursors were then placed in a Teflon-lined stainless autoclave with 60 ml thioacetamide (5 mmol) solution and maintained at 160 °C for 6 h. Finally, the CuCo_2_S_4_ NRAs were obtained after washing and drying. In comparison, the CuCo_2_O_4_ NWAs were obtained by annealing the Cu-Co precursors at 350 °C in air for 2 h.

## Materials Characterization

The phases of the as-synthesized samples were characterized by X-ray diffraction (XRD, X’Pert PRO) with radiation from a Cu target (Kα, λ = 0.154 nm). The surface chemical species of the samples were examined by X-ray photoelectron spectroscopy (XPS, PHI-5000 Versaprobe) from a monochromated Al Kα anode X-ray source (1486.6 eV). The morphologies and microstructures were observed by field scanning electronic microscopy (SEM, FEI Nova NanoSEM 450) and transmission electron microscopy (TEM, FEI Tecnai G2 S-TWIN).

### Electrochemical measurements

The electrochemical properties of the samples were tested in 3 M KOH aqueous electrolytes. All electrochemical measurements were performed in a three-electrode system, where the carbon textile (10 mm × 10 mm) with CuCo_2_O_4_ NWAs and CuCo_2_S_4_ NRAs as the working electrode, Pt foil and saturated calomel electrode (SCE) as the counter and reference electrode, respectively. The cyclic voltammetry (CV) and electrochemical impedance spectroscopy (EIS) tests were carried out on an electrochemical workstation (PGSTAT-302N, Eco Echemie B.V. Company). The EIS measurements were conducted by applying an AC voltage of 10 mV amplitude in a frequency range within 0.01 Hz to 100 kHz. The galvanostatic charge−discharge (GCD) measurements were performed using a CT2001D tester (LAND electronics Co. Ltd., Wuhan, China). Typical mass loading of the CuCo_2_O_4_ NWAs and CuCo_2_S_4_ NRAs was around 1.8 mg cm^−2^ and 2 mg cm^−2^, respectively. The specific capacitance of the as-synthesized samples was calculated from GCD curves with the equation $Cm=I{\rm{\Delta }}t/m{\rm{\Delta }}U$, where I, Δt, m and ΔU are the discharge current (A), discharge time (s), loading mass (g) and voltage drop upon discharging (V), respectively.

### Fabrication of an all-solid-state asymmetric supercapacitor

Prior to the fabrication of the asymmetric supercapacitor, the charges stored in positive (CuCo_2_S_4_ NRAs) and negative (Activated Carbon) electrodes were balanced by calculating the capacitances from the GCD curves in three electrode systems. The total mass of the active material in both electrodes was measured to be about 9.0 mg. The Activated Carbon (AC) electrode was prepared by a simple slurry coating method. Briefly, 80 wt% AC, 10 wt% carbon black and 10 wt% polytetrafluorene-ethylene (PTFE) were homogeneously mixed in DI water, then the slurry was coated onto a piece of carbon textile (10 mm × 20 mm in size) and dried at 80 °C overnight. The PVA-KOH gel electrolyte was obtained by mixing 4 g polyvinyl alcohol (PVA) and 1.63 g KOH in 40 ml of DI water, and heated at 95 °C under stirring until the solution became clear. To fabricate a flexible all-solid-state asymmetric supercapacitor, the as-prepared CuCo_2_S_4_ NRAs and AC electrodes were soaked in the PVA-KOH electrolyte for 15 min and then assembled together with a filter paper as the separator. The energy and power densities were measured by the equations $E=(1/2)C{({\rm{\Delta }}U)}^{2}$ and $P=E/{\rm{\Delta }}t$, where C (F g^−1^), ΔU (V), E (Wh kg^−1^) and P (W kg^−1^) are the mass specific capacitance as a function of the total weight of positive and negative materials, voltage drop upon discharging, energy density and power density of the asymmetric supercapacitor, respectively.

## Electronic supplementary material

Supplementary Information
