# Supplementary material for: Construction of porous CuCo2S4 nanorod arrays via anion exchange for high-performance asymmetric supercapacitor
Source: Sci Rep. 2017 Jul 27;7:6681. doi: 10.1038/s41598-017-07102-1 (PMC5532217; doi:10.1038/s41598-017-07102-1)
Supplement: Supplementary file 1 — Supplementary Information [file 41598_2017_7102_MOESM1_ESM.doc]

Construction of porous CuCo_2_S_4_ nanorod arrays via anion exchange for high-performance asymmetric supercapacitor

Siyi Cheng, Tielin Shi, Chen Chen, Yan Zhong, Yuanyuan Huang, Xiangxu Tao, Junjie Li,Guanglan Liao, and Zirong Tang.

State Key Laboratory of Digital Manufacturing Equipment and Technology,

Huazhong University of Science and Technology

*corresponding author, 1037 Luoyu Road, Wuhan 430074, China. Tel.: +86 27 87792241; fax: +86 27 87792413; E-mail address: zirong@hust.edu.cn (Z. Tang)

**
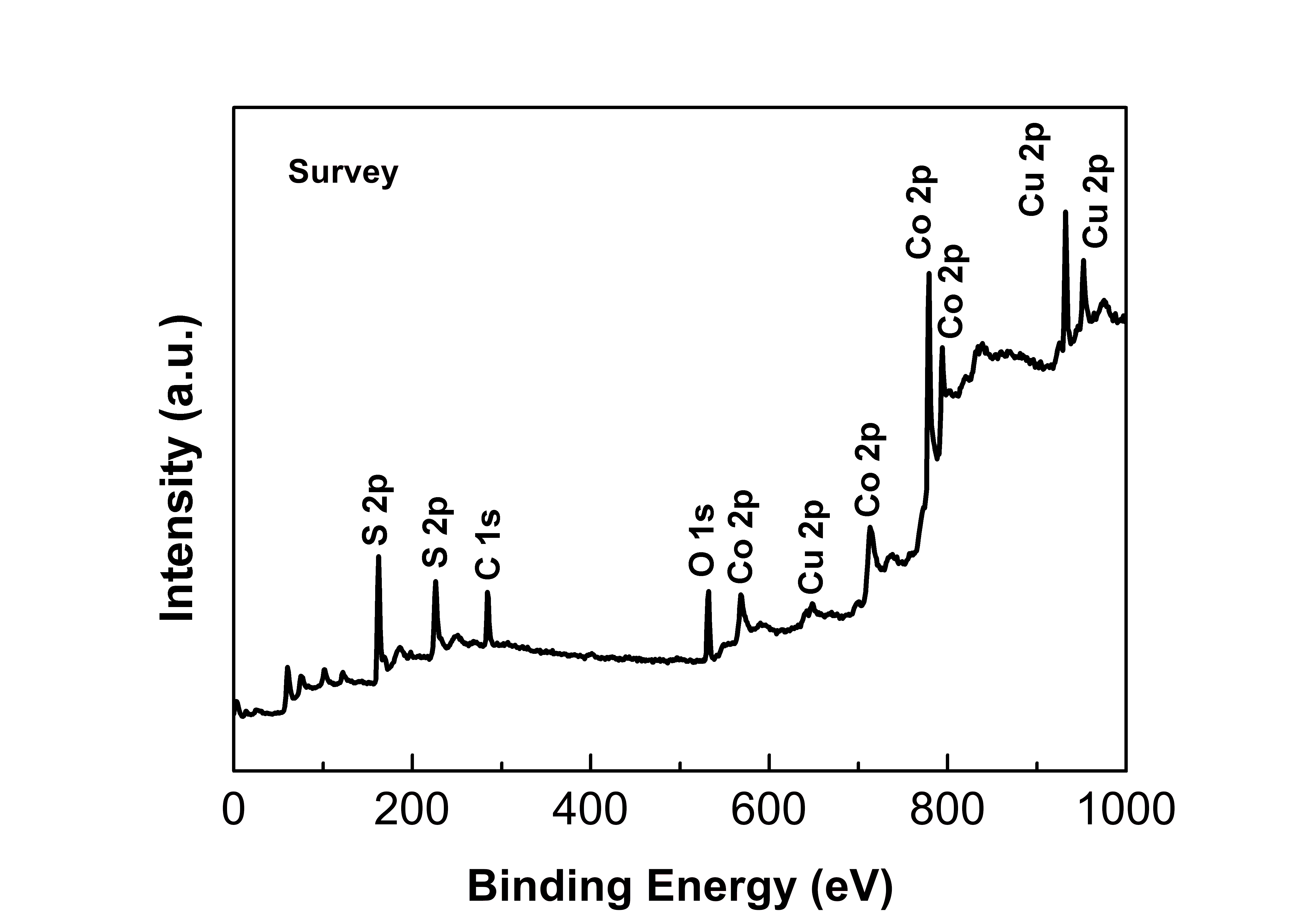
**

**Fig. S1** The typical XPS survey spectrum of the CuCo_2_S_4_ NRAs on carbon textile.


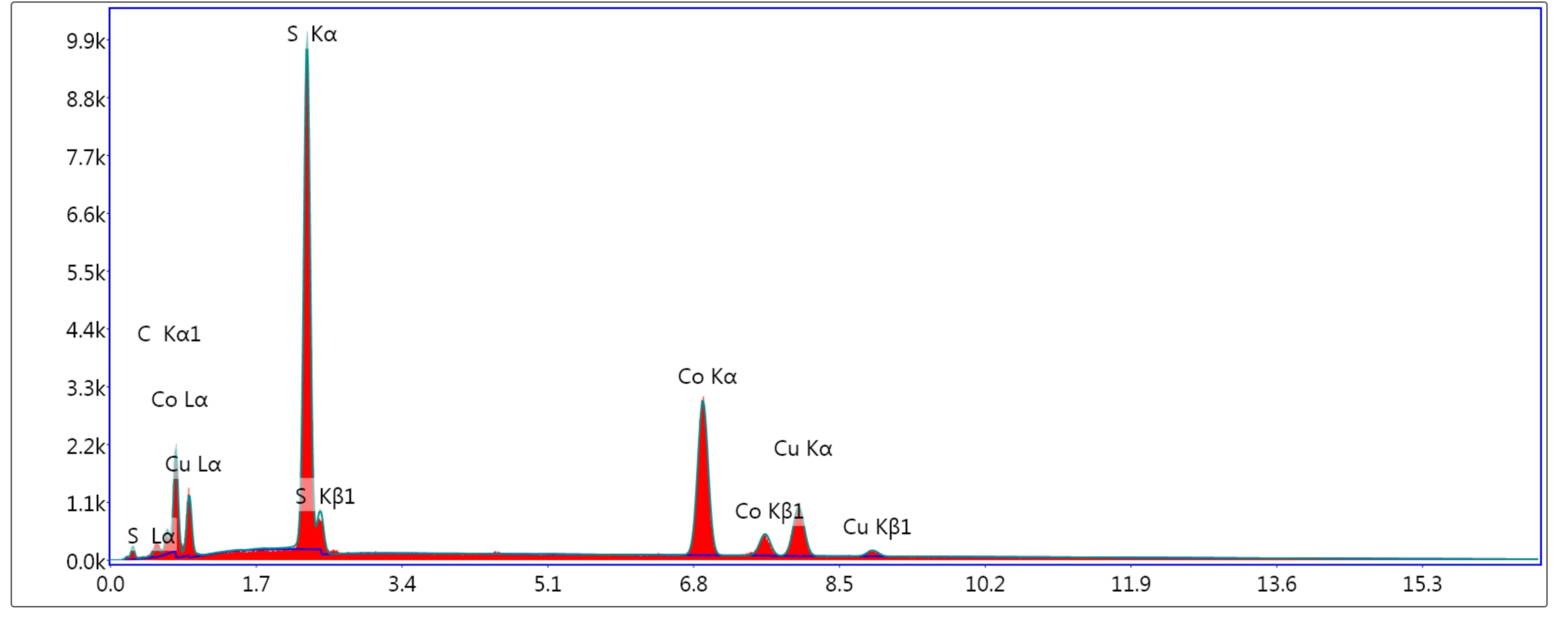
 **Fig. S2** EDS spectrum of the CuCo_2_S_4_ NRAs on carbon textile.


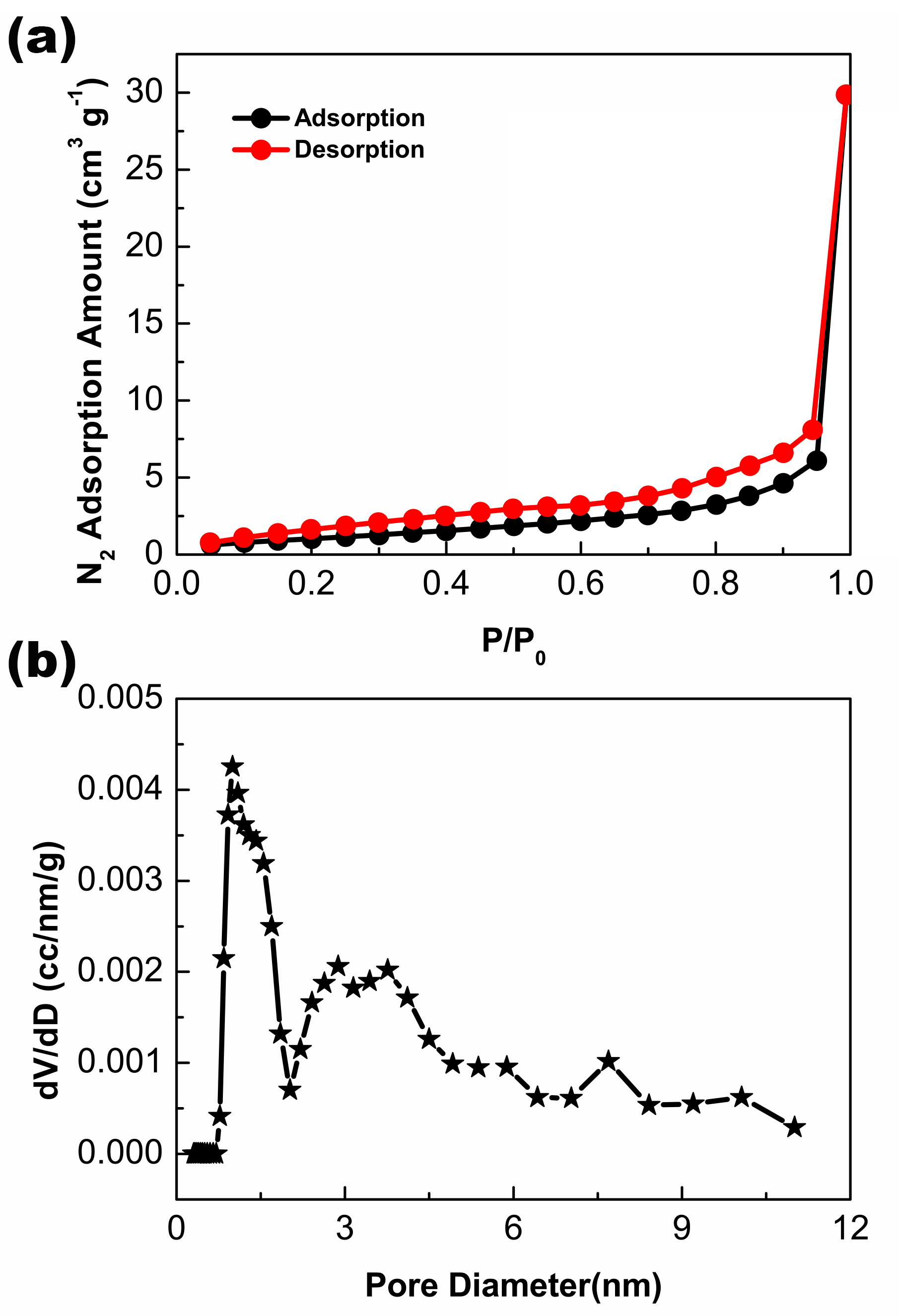


**Fig. S3** (a) Nitrogen adsorption-desorption isotherm of the CuCo_2_S_4_ NRAs. (b) Pore size distribution curve of the CuCo_2_S_4_ NRAs.


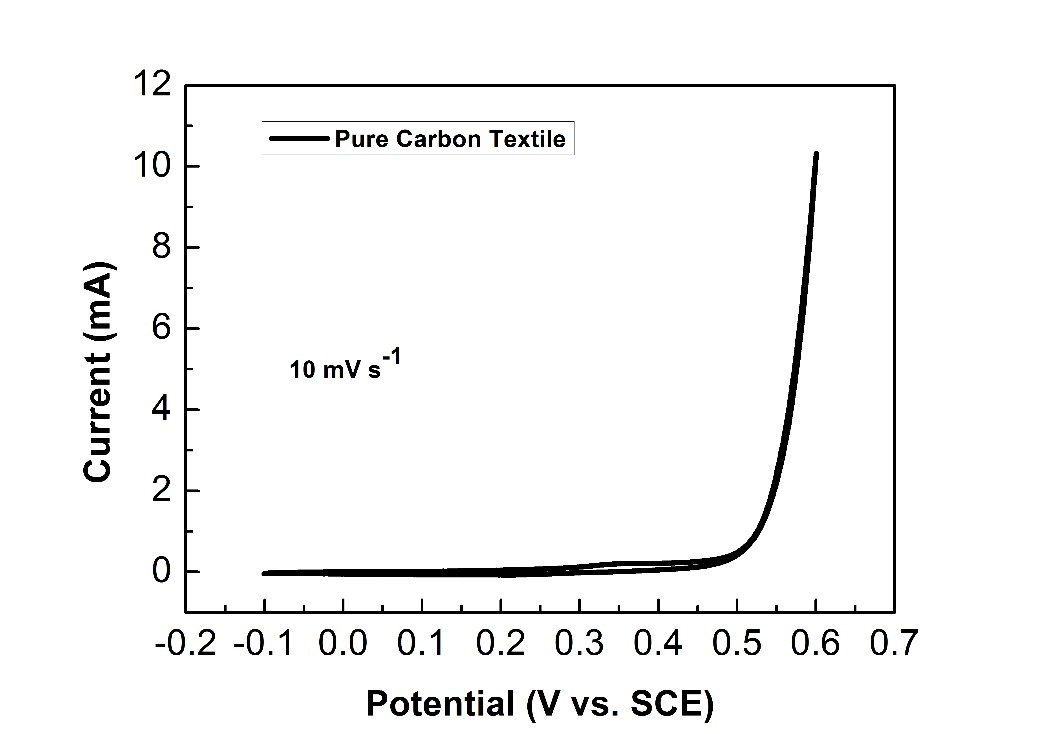


**Fig. S4** CV curve of a pure carbon textile at a scan rate of 10 mv s^-1^ in 3 M KOH.


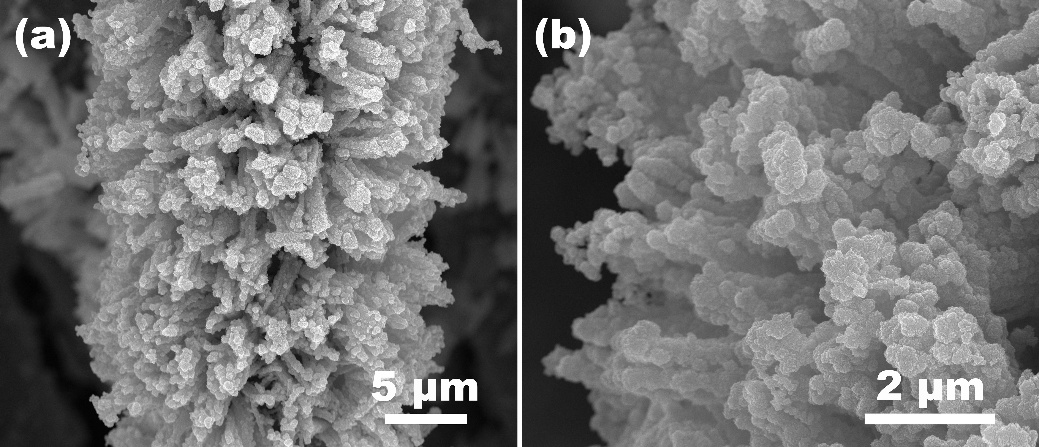


**Fig. S5** SEM images of the CuCo_2_S_4_ NRAs on carbon textile after 10000 cycles.


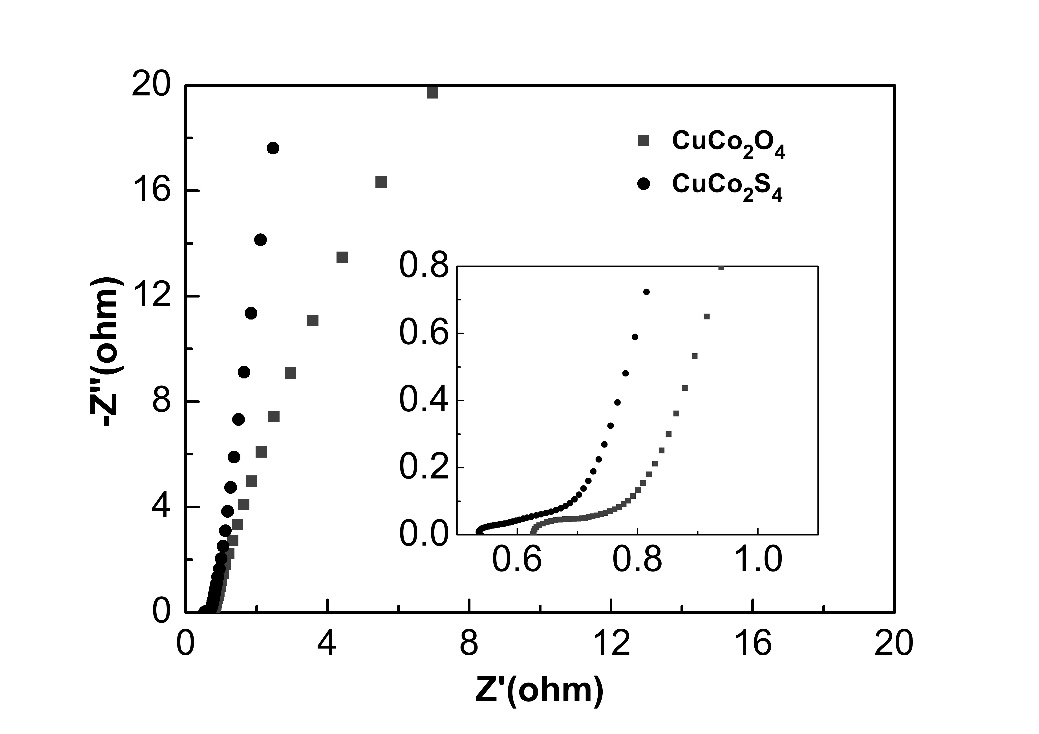


**Fig. S6** EIS plots of the CuCo_2_O_4_ NWAs and CuCo_2_S_4_ NRAs electrodes.
